# Is epistemic injustice a worthy application to mental health nurse education?

**DOI:** 10.1177/09697330241259154

**Published:** 2024-08-09

**Authors:** Jane Fisher

**Affiliations:** 6723 University of Central Lancashire

**Keywords:** mental health nurse education, epistemic injustice, nursing ethics, testimonial injustice, critical psychitary, mental health nursing

## Abstract

This paper explores the philosophical concept of epistemic injustice and contends its significance and relevance to mental health nurse education and clinical practice. The term epistemic injustice may be unfamiliar to mental health nurses, yet the effects are readily visible in the dismissing, silencing, and doubting of service users’ knowledge, testimony, and interpretation. Existing professional values and clinical standards lack depth and critical exploration pertaining to epistemology and associated ethical concerns. Despite central tenets of person-centred care and valuing the service users’ voice, epistemic injustice continues to occur. Epistemic injustice cannot be summed up merely by asking nurses to listen to service users. This represents an oversimplification of epistemology, ignoring the complexities of social influence and knowledge exchanges. Epistemic injustice brings something new and innovative to the nursing curriculum and fits within the principles of heutagogy. It encourages deep reflexivity surrounding the ethical issues of power inequalities and intersectionality. Inclusion in mental health nursing education allows for the social and political powers of psychiatric diagnosis as a form of silencing and stigma to be examined. Practical application is made to mental health nursing education and practice with epistemological values and ethical reflexive prompts. These can be utilised by educators and lecturers for pre-registration mental health nurse education, post-registration, and continued professional development.

## Introduction: What is epistemic injustice?

Epistemic injustice was first introduced by Miranda Fricker^1^ as a philosophical theory pertaining to injustices that occur around knowledge and interpretation. It explicates how certain groups or individuals can be silenced or marginalised in both knowledge transmission and interpretation. The original Frickerian framework of epistemic injustice comprises two district types – testimonial injustice and hermeneutical injustice. Testimonial injustice occurs when an individual’s word, or testimony, is dismissed or disbelieved, based on negative and unfounded prejudices around personal traits or characteristics.^1^ For example, one’s testimony may be doubted because of age, sex, culture, or, importantly, for this paper, mental health condition.

According to Fricker,^1^ hermeneutical injustice occurs prior to testimonial injustice and arises when individuals or groups are denied access to conceptual tools to interpret their experiences. Fricker refers to this lack of interpretative knowledge as a hermeneutical lacuna. It renders those under this lacuna, or gap, with limited knowledge and understanding with which to express their experiences to others. This limits their epistemic power and influence. Both testimonial and hermeneutical injustice are intricately linked to power imbalance and social inequalities. It inhibits the transfer and distribution of knowledge. Those with social power and status are perceived to be valid and credible knowers. Those with less social power are at a risk of receiving unjust deflated levels of credibility.

## Epistemic injustice and health care

Since Fricker’s pioneering work, the theory of epistemic injustice has evolved and developed and been incorporated into a variety of fields, including law, education, and health care. Carel and Kidd^2^ argue that people with medical conditions are vulnerable to epistemic injustice. These originate from power asymmetries within health care systems, alongside cultural stereotypes associated with ill persons. They can be presumed to be cognitively impaired, overemotional, and even existentially unstable.^2^ Crichton et al.^3^ initiated an application to psychiatry, arguing that those with mental disorders (the language used in the paper) are even more vulnerable to epistemic injustice than those with physical health conditions. They identified three contributory conditions for epistemic injustice in psychiatry. These being the impact, or problems caused by mental illness, higher value given to evidence from health care professionals, and entrenched stereotypes associated with mental illness. This initial application of epistemic injustice to psychiatry makes a compelling case that people with mental illnesses are highly vulnerable to experiencing epistemic injustice.^4^

Regrettably, one does not have to look hard to seek patient accounts stating they are ignored, disregarded, and not taken seriously by health care professionals. Lived experience literature around epistemic injustice is vast and rich, describing service users’ experiences of being doubted, silenced, or dismissed.^5^ It is argued that injustices around knowledge and interpretation existed long before Fricker named the phenomena. This paper argues that service users’ and survivors’ experience and accounts of epistemic injustice have existed long before psychiatrist and academics ‘applied’ the theory to psychiatry. Service user and survivor descriptions of being inter alia dismissed and ignored by mental health nurses predate this academic adoption of the phenomena of epistemic injustice. The oppression and discrimination of mental health service users has long been documented by psychiatric survivors. The intent of this paper is not to further perpetuate epistemic injustice by co-opting these accounts or claiming coinage of new nursing phenomena and associated intervention. The aim is to amplify these voices and experiences of injustice, including the author’s personal experiences of epistemic injustice as a service user. The intention of this paper is to elevate the damaging and traumatic effects of epistemic injustice, to allow for preventative and ameliorative practices to be woven into mental health nurse theory, practice, and education.

The theory of epistemic injustice has vastly expanded since its introduction by Fricker.^1^ Alongside testimonial and hermeneutical injustice is a rich and varied body of the literature and discourses considering epistemic injustice against varied backdrops such as research,^6^ coproduction,^7^ advocacy,^8^ mental health legislation,^9^ and specific psychiatric diagnoses, for example, personality disorder.^10^ There are multiple subtypes of epistemic injustice,^11^ including pre-emptive epistemic injustice where patients are not even consulted, despite them having epistemically relevant knowledge. This is explored by Bueter^12^ in relation to psychiatric classification. However, for this paper, the term epistemic injustice is used as an umbrella term to denote epistemological wrongs associated with mental illness. This is a limitation of this paper; however, the purpose is to make the initial argument to include in the nursing curriculum. As the application is unexplored within mental health nursing education, further nuances can be explored in subsequent literature, alongside more focused application to teaching and learning, in line with the principles of heutagogy. Much academic application to date has focused on philosophical and ethical debates, or the role of the individual psychiatrist. With nurses forming the largest number of clinicians providing mental health care, this paper argues for the relevance of epistemic injustice within mental health nursing education and clinical practice. This is unexplored in the literature to date.

## Mental health nurse education

Mental health nurse education often has an equal split between theory delivered at Higher Education Institutes (HEIs) and practical placements supervised by registered nurses. Mental health nursing curriculums are already saturated in terms of theory hours, with educators forced to make problematic decisions on what theory content to include. It is a challenging task to request additional theory time dedicated to an academic philosophical theory that may not be familiar to educators themselves. However, this paper will demonstrate that principles of epistemic injustice can be woven into existing content, for example, discussions about advocacy, non-discriminatory practice, stigma, coercion, and person-centred care. This will give a greater depth to the criticality and reflexivity of future mental health nurses. It sits well within principles of heutagogy and the creation of independent critical learners.

## Do existing professional standards encompass epistemic injustice?

When considering the value of adding epistemic injustice to mental health nurse education, one must determine if existing standards prevent injustices around knowledge exchange. In the UK, the Nursing and Midwifery Council (NMC) outlines professional standards and core values for registered mental health nurses, alongside regulation for pre-registration nursing education.^13,14^ Central tenets of the NMC Code embody respect, person-centred care, advocacy, and listening to patients, all which have a role in the prevention of epistemic injustice. Similar tenants are reflected globally. Connell et al.^15^ theorise that a philosophical shift in the UK resulted in the dilution of advanced mental health nursing skills in the NMC 2018 updates. Warrender^16^ concurs that current mental health nurse education does not equip nurses for the complexities of clinical practice. This is a wide-reaching problem with the literature from Australia questioning whether current mental health nurse education meets the needs of consumers of mental health care.^17^

One may question if the central tenants of universal mental health nursing values encompass the theory of epistemic injustice and are enough to prevent the epistemological wrongs experienced by service users? The vast and rich literature crafted by service users and survivors would suggest otherwise. Incidents of injustice remain despite simplistic instructions to ‘actively listen’, suggesting a more comprehensive approach is needed, strengthening the case for inclusive in the nursing curriculum. Connections between professional standards^13,14^ and epistemic injustice are at best loose, and at worst sloppy. Such vague and one-dimensional instructions neglect to address the intricacies of mental health nursing care and ethical predicaments around knowledge exchange.

Epistemic injustice brings a rich depth to naïve directions and assertions to ‘listen to’ patients. However, Kious et al.^18^ argue that the theory of epistemic injustice adds nothing beyond what good standards of care require. This is centred on individual interactions between psychiatrists and service users. It is beyond the scope of this paper to investigate clinical standards of care for psychiatrists; however, one may posit that clinical standards are as equally vague and unsophisticated as nursing standards.

## Does epistemic injustice unnecessarily overcomplicate mental health nurse education?

As alluded to in the abstract for this paper, epistemic injustice maybe an unfamiliar term to mental health educators, academics, nurses, and student nurses. However, when the effects of epistemic injustice are explored with student nurses, the author notices a ‘light bulb’ moment, where students can identify moments from clinical practice which could be interpreted as epistemic injustice. Further empirical research is needed to explore student nurses’ opinions about the added value of epistemic injustice to their education and clinical practice. More research is needed on how to successfully implement epistemic injustice into pre-registration nursing education pedagogy.

Mental health nurses operate within complex ethical realms, frequently enforcing ‘treatment’ against someone’s will, whilst simultaneously claiming to be providing compassionate nursing care. This paper contents that mental health nursing values and education warrant complication and deep ethical reflexive examination, under the principles of epistemic injustice. No apology is made for inciting nurses and educators to deliberate deeply about their personal embodiment of nursing values and how they navigate moral predicaments. Mental health nurse education urgently needs philosophical and ethical exploration, in line with principles of heutagogy and creating learners who are independent critical thinkers.

## Does epistemic injustice add anything new to mental health nursing theory and practice?

It has already been established that professional standards for mental health nursing are deficient in considering the complex role of epistemic injustice and its impact on service users and survivors. Including epistemic injustice in nurse education does not unnecessarily overcomplicate nursing education. This paper will now argue that epistemic injustice brings something original and innovative to mental health nursing education and clinical practice that is currently lacking. This will be beneficial for service users and the mental health nursing workforce.

## Power imbalances

Epistemic injustice can offer a way to understand some nuances associated with why service users can feel dismissed and silenced, both from within and outside of mental health services. This can enhance nurses’ insight and comprehension of the injustice and oppression pertaining to knowledge exchange. By encouraging mental health nurses to reflect on whose knowledge and testimony is given credibility, and who is dismissed, creates a space for deep and worthy considerations of social powers. Considering these power imbalances promotes deep reflexivity.

Epistemic problems within health care are woven into the fabric of medical institutions. This systemic and institutional deafness to the voice of the patient is evident in the language used in health care.^19^ This can indicate collective negative attitudes towards patient perspectives, indicative of power imbalances. According to Sibley, patient feedback is frequently labelled as anecdotal evidence. This implies a lack of reliability, objectivity, and accuracy. This is further compounded by the rhetoric embedded within health care where staff complete ‘incident forms’, whereas patients make ‘complaints’. The language of complaints is highly negatively charged, in contrast to ‘nursing records’. This rhetoric cements the epistemic authority of the clinician. It is of vital importance that mental health nurses are acutely aware of the credible and trustworthy authority attributed to them, granting epistemic power.

Russo and Beresford^20^ argue service user (or mad) voices are overwritten, silenced, and even erased under the domination of the medical model. The 21^st^ century mental health nurse needs to consider these complex interactions and their role in the silencing and dismissal of the service user’s voice. If students are open to a service users’ story, their personal narrative, and distinct meaning making, hermeneutical (interpretative) knowledge is created. This would allow for what Sagan^10^ advocates in separating someone’s story from the influence and domination of diagnostic criteria and the medical model.

## Psychiatric diagnosis

An additional value to mental health nurses, academics, and educators is the encouragement to think beyond individual interactions with service users and consider the wider social and political context of psychiatry. Stigma and negative stereotypes are quoted by Crichton et al.^3^ as being a reason for mental health service users experiencing and being vulnerable to epistemic injustice. However, what this viewpoint does not consider is that psychiatric diagnosis itself is a form of epistemic injustice and oppression.^21^

Mental health nurses have a colossal role to play within the medical model of psychiatry (despite claims of a holistic approach). They regularly partake in enforced treatment and the use of legal powers associated with psychiatric diagnosis. Considering this from a perspective of epistemic injustice encourages student nurses to consider the wider ethical implications of the hegemonic medical model. Mental health nursing does not have the monopoly on mental distress and nurses require both humility and criticality to consider alternative explanations for distress, alongside political influences on health care. Including epistemic injustice in mental health nurse education will allow for additional considerations of intersectionality and how injustices around knowledge and interpretation are imbued with other factors such as race, gender, and social class. Epistemic injustice is a nexus to diagnostic overshadowing allowing reflection on scenarios where service users’ physical health complaints are dismissed based on their status as a mental health patient.

Mental health service users, or avoiders,^22^ have been documenting their traumatic experiences of being dismissed by mental health professionals when seeking help for suicidal thoughts. This can be interpreted as epistemic injustice because of the diagnosis or assumed diagnosis of emotionally unstable personality disorder, or the latest lexicon evolution, complex emotional needs.^5,23^ This contested diagnostic label brings entrenched systemic stigma from mental health professionals. When seeking help for suicidal thoughts, individuals are frequently dismissed and disbelieved, based on a perverse concept that if one was truly suicidal, they would not seek help.^24,25^ Again this is evidence of service users falling victim to testimonial injustice, and their testimony is disregarded because of unfounded prejudice against a psychiatric diagnosis. This entrenched stigma from within mental health services, and because of psychiatric diagnosis, is far more nuanced than the societal stigma described by Crichton et al.^3^

Having considered the added value of epistemic injustice to mental health nurse education and establishing that this is not fulfilled by professional standards, this paper will now consider two critiques of epistemic injustice. The first being service users’ expectations of care, which is easily refuted. The latter, warning of the risk of co-option by academia which warrants a more considered and nuanced discussion.

## Will calls for epistemic injustice give service users unrealistic expectations of mental health care?

This is a weak criticism against calls for epistemic injustice to be contemplated within psychiatry. Kious et al.^18^ make an audacious claim that an overemphasis on epistemic injustice could cause significant harm to service users and their relationships with mental health services. They posit that epistemic injustice could lead to unrealistic expectations of care, where patients would assume their opinions and views to be unquestionably accepted. This is a provocative assertion, based on an unfounded understanding of true epistemic injustice. There is no obligation to remove all clinical judgement and epistemic sensitivity in the clinical encounter.

This critique is easily dismissed as no proponents of epistemic injustice, nor this paper, advocate that clinicians unquestionably believe everything a service user says and forgo clinical judgement, critical thinking, and professional curiosity.^26^ When there is what Crichton terms ‘good reason’ to doubt someone’s testimony, this should be investigated. Good reason could be a known and previously confirmed delusion. These incidents of temporary irrationality within in mental health care illuminate the complexities that mental nurses navigate in the realm of knowledge exchange.

This argument against the significance of epistemic injustice ignores the complexities of epistemology and mental illness. A focus on individual clinician encounters disregards the wider universal impacts of knowledge exchange within complex social and political realms. It forgoes the systemic impact of psychiatric diagnosis as, in fact, a perpetrator of epistemic injustice.

Although hopefully unintentional, it also overlooks the intelligence of mental health services users. Being simultaneously a mental health nurse academic and service user gives me a dual vantage point of reflection. I have been caused considerable damage by mental health services, ignoring my authentic testimony. The expectation of being listened to, heard, and understood is by far not an unrealistic expectation that anyone should expect of health care professionals.

However, I most certainly do not expect mental health professionals to unquestioningly believe at face value everything that I say or request, for fear of perpetuating epistemic injustice if they do not. Such a claim both undermines my intelligence and neglects to consider the nuances of mental distress and rationality. This paper argues that this audacious critique of epistemic injustice in fact perpetuates epistemic injustice and reflects a stigma and presumption that those of ‘them’ with mental illness are unreasonable and foolish. I have made requests to mental health professionals that I am wholeheartedly glad they did not simply ‘believe’ at face value, because the results would be tragic. What I need in these moments are skilled mental health professionals who known me and can discern temporal irrationality triggered by mental distress, from my authentic self. I expect them to respectfully hear my narrative and try to understand my perspective – even if this is temporarily influenced by illness.^27^ There is value and meaning (perhaps more so) in the realms of psychosis.

## Is there a danger of co-option by mental health academia?

This critique of epistemic injustice requires considered reflection by academics, and those of us occupying a place in both academia and psychiatric survivor communities. Although resonating as empowering with some mental health service users or psychiatric survivors,^23,28^ the theory of epistemic injustice is not without critique. Russo and Beresford^20^ fear service user narratives and personal accounts are at a risk of being colonised by academia. This would further perpetuate epistemic injustice and associated harms, by taking service user accounts and re-labelling them as epistemic injustice. This represents a challenge when exploring epistemic injustice within academia and nurse education. This paper advocates for the importance of learning via lived experience^29^ with the addition caution of avoiding co-option and reinterpretation. This requires a nuanced and multifaceted approach to co-production in education. More recently Russo^21^ argues that epistemic injustice is debated more by clinical academics than people with lived experience of mental illness or the mental health system. There is a risk the concept is over intellectualised, thus becoming inaccessible to the people experiencing the injustices and associated oppression.

The risk of co-option by academia is a legitimate cause for concern. Caution must be applied when taking a principle aimed at the empowerment of service users and using it for academic status and credibility. Historically, the dangers of co-option can be illustrated by the recovery movement. Primarily initiated by grassroots survivors,^30^ the noble message of recovery was eventually co-opted by neoliberal mental health services and used as a social and political mechanism. One example being the ‘back to work’ agenda.^31^ Additionally, service users (including myself) have felt the pressures described by Sagan (2020)^10^ to conform our personal narrative to idealised expectations of recovery, for example. The fate of resilience is currently hanging in the balance. Again, despite aims to empower service users, it has been interpreted as a personal quality or strength, used to place individual responsibility onto service users, thus disregarding social and political expectations.^32^

The above represents the challenges associated with including service user’s experiences of epistemic injustice in mental health nurse education. The challenge for lecturers and educators is to welcome all people’s experiences of mental illness, distress, or however they make sense of their experiences. There is a risk of recruiting people who have a positive experience of mental health care, thus excluding anyone with a different narrative. However, to create critical mental health nurses, they need a variety of peoples’ experiences outside of the idealised, re-crafted, edited versions of apparent recovery.

## Practical application to mental health nursing education

This paper is not an unrealistic call for HEIs adding additional modules to their already saturated mental health curriculums. However, this paper contents that there are various opportunities when discussing person-centred care, stigma, advocacy, family involvement, co-production, non-discriminatory care, and coercion to include some short additional reflections based upon epistemic injustice. The intention is not to be simplistic and one dimensional, as this has already been illustrated as woefully inadequate. The aim is to provoke reflexivity in mental health nurses, students, and educators. This paper proposes the following considerations and reflections that can be woven into existing curriculum content.

• Honour and embrace the voice of the service user, taking time to hear their story, and importantly, the sense they make, and the individual interpretations of their experiences and life story. Adopt professional curiosity and an open mind, seeking to listen and understand, not to reinterpret or re-write.
• Employ humility. As nurses, we do not have all the answers. Psychiatry is lacking in definitive interpretations and infallible solutions to emotional distress. No single explanation or discipline has the monopoly on mental illness or distress. Not only be open to alternatives outside of the medical model but also actively seek them out broadening your expertise.
• Be self-aware and reflective, examining oneself for unconscious bias concerning individual service users or diagnoses. Challenge stigma within oneself, others, or teams, remaining non-judgemental and leading with integrity and authenticity.
• The epistemic privilege granted to us as mental health professionals brings significant power and authority. An awareness of the potential for domination and silencing is vital for a critical mental health nurse. With deep reflexivity examine the impact of power imbalances within mental health care and the role of the nurses in perpetuating epistemic injustice.
• Reflect on intersectionality and the multiple factors of oppression that impact individuals. For example, race, gender, and social class all add another dimension to discrimination caused by mental illness. Consider how these factors interact with each other for the individual.
• Investigate someone’s testimony should there be *good reason* to doubt someone’s credibility. Do this with sensitivity and respect.
• What can you learn from service users? What can they teach you about the lived experience of mental illness that textbooks cannot?
• Advocate for service users and their loved one’s rights and wishes. Support service users to feel seen and heard in a system based on power imbalances with professionals’ testimony automatically given precedence. Empower service users and their loved ones by amplifying their voice.
• Reflect on scenarios where you can identify epistemic injustice as a contributing factor to testimony or explanations being dismissed, silenced, or not believed. Consider the impact on services users and their loved ones. How could you approach this differently to achieve epistemic justice? What are the challenges of achieving epistemic justice in clinical nursing practice?

## Conclusion

To conclude, this paper has made an argument for including epistemic injustice in mental health nurse education and professional development in alignment with the principles of heutagogy. Existing nursing values and professional standards do not adequately incorporate epistemic injustice and the associated nuances. A true embodiment of epistemic injustice in mental health nursing (or any other mental health professional) goes beyond simplistic calls for person-centred care and listening to service users. There is added value when considering epistemic injustice within mental health nursing theory and practice. It brings deep reflexivity around power imbalances and the role of psychiatric diagnosis. Two critiques of epistemic injustice have been considered, with a warning against the academic co-option of colonisation of epistemic injustice. Finally, a practical application is made to mental health nursing with reflexive considerations that can be incorporated into the existing mental health nursing curriculum to aid ethical discussion and the creation of critical mental health nurses.
